# Exploring the Correlation Between Lipid Packaging in Lipoplexes and Their Transfection Efficacy

**DOI:** 10.3390/pharmaceutics3040848

**Published:** 2011-11-18

**Authors:** Behfar Moghaddam, Sarah E. McNeil, Qinguo Zheng, Afzal R. Mohammed, Yvonne Perrie

**Affiliations:** School of Life and Health Sciences, Aston University, Birmingham, B4 7ET, UK

**Keywords:** Langmuir studies, lipid packaging, cationic liposomes, gene delivery, transfection, electrolytes

## Abstract

Whilst there is a large body of evidence looking at the design of cationic liposomes as transfection agents, correlates of formulation to function remain elusive. In this research, we investigate if lipid packaging can give further insights into transfection efficacy. DNA lipoplexes composed of 1,2-dioleoyl-*sn*-glycero-3-phosphoethanolamine (DOPE) or 1,2-distearoyl-*sn*-glycero-3-phosphoethanolamine (DSPE) in combination with 1,2-dioleoyl-3-trimethylammonium-propane (DOTAP) or 1,2-stearoyl-3-trimethylammonium-propane (DSTAP) were prepared by the lipid hydration method. Each of the formulations was prepared by hydration in dH_2_O or phosphate buffer saline (PBS) to investigate the effect of buffer salts on lipoplex physicochemical characteristics and *in vitro* transfection. In addition, Langmuir monolayer studies were performed to investigate any possible correlation between lipid packaging and liposome attributes. Using PBS, rather than dH_2_O, to prepare the lipoplexes increased the size of vesicles in most of formulations and resulted in variation in transfection efficacies. However, one combination of lipids (DSPE:DOTAP) could not form liposomes in PBS, whilst the DSPE:DSTAP combination could not form liposomes in either aqueous media. Monolayer studies demonstrated saturated lipid combinations offered dramatically closer molecular packing compared to the other combinations which could suggest why this lipid combination could not form vesicles. Of the lipoplexes prepared, those formulated with DSTAP showed higher transfection efficacy, however, the effect of buffer on transfection efficiency was formulation dependent.

## 1. Introduction

In the development of new healthcare strategies, correction of underlying pharmacology of diseases instead of alteration of symptoms remains a key goal. Gene therapy has been developed with this aim in hand and both genetic diseases and acquired diseases are two main groups of diseases which gene therapy could play an important role in their treatment [1]. Yet developments in gene therapy remain slow and the major unresolved concern with gene therapy is how to effectively deliver the gene to the target site. Although it has been known for some time that direct injection of ‘naked’ DNA allows transgene expression in muscle [2], in most of the cases ‘naked’ DNA molecules are not able to enter cells efficiently due to their large size, negative charge and nuclease mediated degradation *in vivo* [3]. Therefore, a delivery vehicle (vector) must be used to carry the gene into the target cell or tissue to save the gene from the above dangers.

An ideal vector should be stable and have high efficiency, with minimal toxicity and unrestricted size limitation for genetic payload (which can include DNA, siRNA *etc.*). It should also be easy to prepare and be cost effective [4]. Amongst the two classic groups of vectors, viral vectors provide high transfection efficiency but these systems are still limited by the dogma of toxicity and immunogenic reactions and therefore much research has focused on the use of non-viral vectors, given their superior safety profile despite their lower efficacy. In the case of non-viral systems cationic liposomes, due to their ability to address several of the above criteria, has been widely investigated [5–10] and the electrostatic interaction between positive charge of cationic liposomes and negative charge of DNA make a complex of cationic liposome-DNA generally referred to as lipoplexes [11].

To develop lipoplexes with high transfection and low toxicity, several parameters should be taken into account such as: size, lipid/DNA charge ratio, net positive charge of the lipoplex, chemical structure of cationic lipid and helper lipid, and finally the structure of the complex itself [12]. However given the dynamic nature of these structures many factors, in addition to those already listed, can contribute to the resultant physico-chemical attributes of the lipoplex including the rate of mixing of the various components, the temperatures used and even the presence of electrolytes in the buffers used [12–14]. Indeed recent research has shown that the presence of electrolyte within the aqueous media can influence both the physico-chemical properties and the *in vivo* efficacy of lipoplexes, with the authors demonstrating that the addition of low concentrations of sodium chloride to cationic liposomes during complex formation lead to an improved vaccine adjuvant action [15,16].

**Figure 1.**
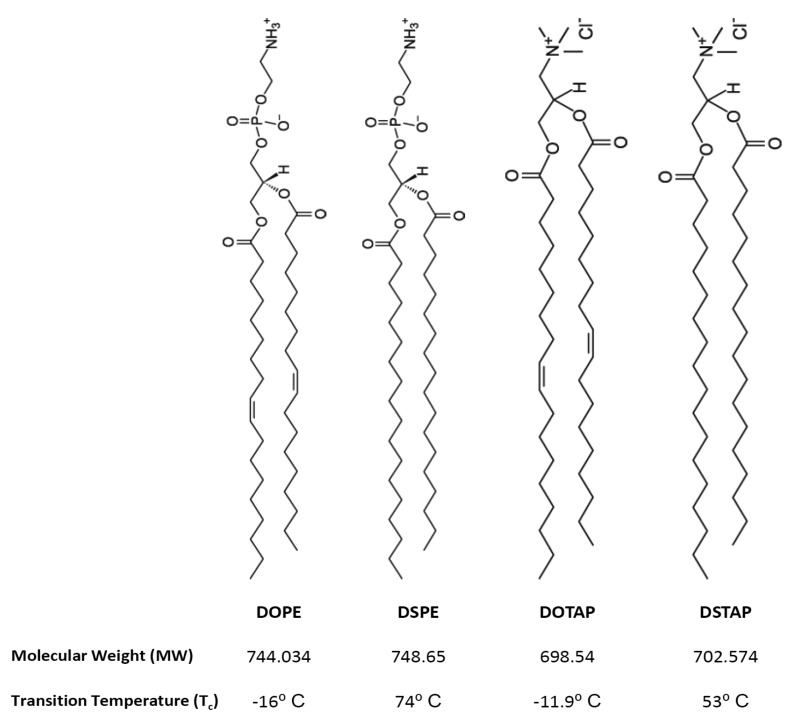
Molecular structure of DOPE and DSPE and the cationic lipids DOTAP and DSTAP used. (DOPE: 1,2-dioleoyl-*sn*-glycero-3-phosphoethanolamine, DSPE: 1,2-distearoyl-*sn*-glycero-3-phosphoethanolamine, DOTAP: 1,2-dioleoyl-3-trimethylammonium-propane, DSTAP: 1,2-stearoyl-3-trimethylammonium-propane).

Thus the aim of this research was to investigate the molecular interactions of lipids and the resultant lipoplexes properties and to attempt to correlate these with the transfection attributes of the system in a controlled *in vitro* environment. Therefore lipid monolayers were studied by Langmuir-Blodgett trough as such monolayers can be considered as building blocks for bilayer vesicles, consequently, studying these monolayers in an aqueous media (dH_2_O or PBS) at air/water interface, in combination with the lipid attributes may give insights into bilayer lipid packaging configuration and liposome stability which could influence transfection [17]. Cationic liposomes were prepared in dH_2_O or the commonly used phosphate buffered saline (PBS) and the liposome physico-chemical characteristics considered such that the effect of electrolytes could be considered and correlated with the Langmuir studies. Of the cationic liposome systems tested, the combination of the fusogenic lipid DOPE with the cationic lipid DOTAP, is a frequently used composition due to its high *in vitro* transfection efficiency and optimal immune response [11,18–22]. Therefore this was chosen for further investigation. To consider the effect of lipid acyl chains on their molecular packaging and lipoplex characteristics were also systematically compared using DOPE and DOTAP with their disteroyl equivalents (DSPE and DSTAP) (Figure 1).

## 2. Materials and Methods

### 2.1. Materials

1,2-Dioleoyl-*sn*-glycero-3-phsphoethanolamine (DOPE), 1,2-dioleoyl-3-trimethylammonium-propane (DOTAP), 1,2-stearoyl-3-trimethylammonium-propane (DSTAP) and 1,2-distearoyl-*sn*-glycero-3-phosphoethanolamine (DSPE) were purchased from Avanti Polar Lipids, Inc. (Alabaster, AL). Ethylenediaminetetraacetic acid (EDTA) and phosphate buffer saline (PBS) tablets were purchased from Sigma-Aldrich Company (Poole, UK). Ethanol, methanol and chloroform (all HPLC grade) were purchased from Fisher Scientific (Leicestershire, UK). PicoGreen^®^ and Lipofectin™ reagents were obtained from Invitrogen Life Technologies and the luciferase assay kit and CellTiter 96^®^ AQ_ueous_ One Solution Cell Proliferation Assay were both obtained from Promega (Madison, WI). Serum free and antibiotic free medium (opti-MEM), Dulbecco's modified Eagles medium (DMEM), L-glutamine/Penicillin-Streptomycin and foetal bovine serum (FBS) were purchased from Gibco-Invitrogen Ltd. (Paisley, UK). gWiz™ Luciferase was obtained from Genovac GmbH, Germany. The African green monkey kidney cells (COS-7 cells) were purchased from European collection of cell cultures (ECACC) a Health Protection Agency Culture Collection (Salisbury, UK).

### 2.2. Liposome Preparation

All cationic liposomes were prepared by the lipid hydration method based on the work of Bangham *et al.*, in 1965 [23]. Briefly the required lipid mixture of a helper lipid and a cationic lipid, at (8:8) μM ratio were dissolved in chloroform:methanol (9/1 v/v) in a round bottom flask and a dry lipid film produced by rotary evaporation at 37 °C. Any residual solvent was removed from the final dry lipid film by a stream of oxygen-free nitrogen. The dried lipid films were suspended in distilled water or PBS as appropriate and vortexed vigorously until all of the lipid film came into suspension to produce multilamellar vesicles (MLV), which were subject to sonication (1 minute at 5 amplitude microns) to produce small unilamellar vesicles (SUV).

### 2.3. Preparation of Cationic Liposome/DNA Complexes (Lipoplexes)

Cationic liposome-DNA complexes were prepared by adding different ranges of plasmid DNA content (2.5, 25, 50, 100, 200 and 1600 μg) and incubated at room temperature for 30 minutes. To perform *in vitro* studies, lipoplexes was prepared by diluting 17.5 μL SUV solution to 0.35 mL with opti-MEM, which was then incubated for 40 minutes at room temperature. After incubation, 0.35 mL of opti-MEM containing 3.5 μg plasmid DNA was added, and gently mixed with SUV solution and incubated again for a further 15 min at room temperature. The resultant SUV-DNA mixture was then diluted to a final volume of 3.5 mL with opti-MEM.

### 2.4. Determination of Lipoplex Size and Zeta Potential

The z-average diameter of lipoplexes was determined by dynamic light scattering using the photon correlation spectroscopy (PCS) technique measured on a Malvern Zetasizer Nano-ZS (Malvern Instruments Ltd., UK). The zeta potential (an indirect measurement of the vesicle surface charge) of the complexes was measured on Malvern Zetasizer NAni-ZS (Malvern Instruments Ltd., UK) at 25 °C in distilled water or PBS as appropriate.

### 2.5. DNA Association Within Lipoplexes

To measure the association of the DNA with liposomes, the preparation was centrifuged (Optima™ Max-xp Ultra Centrifuge; Beckman Coulter, USA) for one hour with the speed of 125,000 × *g* at 4 °C. After centrifugation, the supernatant were collected and the pellets were resuspended in appropriate media and centrifuge repeated for second time. By adding 100 μL PicoGreen^®^ as a fluorescent dye to the 100 μL of supernatants in a 96 well plate and incubation of them up to 5 minutes at room temperature, the absorbance was read by SpectroMax Gemini EM (Molecular Device) plate reader, the amount of non-incorporated DNA was measured.

### 2.6. In Vitro Transfection of COS-7 Cells

African green monkey kidney cells (COS-7 cells) were maintained at 37 °C under 5% CO_2_ in Delbecco's modified Eagles medium (DMEM) with 4 mM L-glutamine and supplemented with 10% (v/v) foetal bovine serum (FBS), penicillin (100 U/mL) and streptomycin (100 μg/mL). COS-7 cells were plated 24 hours prior to transfection, at a cell concentration of 1 × 10^5^ cells/mL in 1 mL of medium in a 12-well plate and incubated overnight. Prior to transfection, cells were washed with 1 mL of opti-MEM before lipoplexes were added to the cells. 1 mL of the SUV-DNA solution 0.0078 μmole total lipid content containing 1 μg plasmid DNA) was added to each well, each in triplicate. After 5 hours incubation at 37 °C in 5% CO_2_, the medium was replaced with growth medium (DMEM) containing 10% FBS and the cells were incubated for 48 hours. Transfection efficiency of each formulation was determined by measuring the percentage of each sample to the control. In this study this value is reported as luciferase activity and Lipofectin was the transfection reagent.

### 2.7. Cytotoxicity Study

Cos-7 cells were pippeted on a 96-well plate and incubated for 24 hours at 37 °C. Then 20 μL of MTS reagent (CellTiter 96^®^ AQ_ueous_ One Solution Cell Proliferation Assay) was added to each well. Cells bioreduce the MTS reagent into a red formazan product. Plates were incubated for 4 hours at 37 °C, in a 5% humid CO_2_ atmosphere. After that the quantity of produced formazan was measured on microplate reader Thermo Scientific Molecular Spectrum plate reader at A_490_. The absorbance reading is directly proportional to the number of living cells in the medium. In this study cell viability is calculated by comparing the results to the positive control (*i.e.*, cells and medium) and expressed as a percentage.

### 2.8. Langmuir Studies

To investigate the surface pressure of monolayer lipids Langmuir-Blodgett technique has been used. It is an automated controlled film balance apparatus (KSV Langmuir Mini-trough, KSV Instruments Ltd., Helsinki, Finland) equipped with a platinum Wilhelmy plate. This instrument was used to collect the surface pressure-area isotherms. The trough was filled with deionised water or PBS solution as appropriate. The substances were prepared at fixed total concentration of 1 mg/mL of lipid in chloroform. 20 μL of solution was dropped on to the air/water interface with a Hamilton micro-syringe. The monolayer was left for 15 min to allow chloroform to evaporate. Then constant rate compression of 10 mm/min was performed on the monolayer molecules until collapse of the monolayer lipid. An external water bath circulation system maintained constant temperature of 20 °C for the subphase. To analyse the data KSV software (KSV Instruments Ltd., Helsinki, Finland) was used.

### 2.9. Statistical Analysis

Means and standard deviation were calculated for all experiments. The one-way analysis of variance (ANOVA) was performed on all data to determine statistical significance. The statistical significance determined to 0.05 confidence intervals (P < 0.05). To compare the difference of significance of different conditions Tukey's post hoc test was performed.

## 3. Results

### 3.1. Molecular Packaging of Lipids: The Role of Lipid Structure and Electrolytes

**Figure 2.**
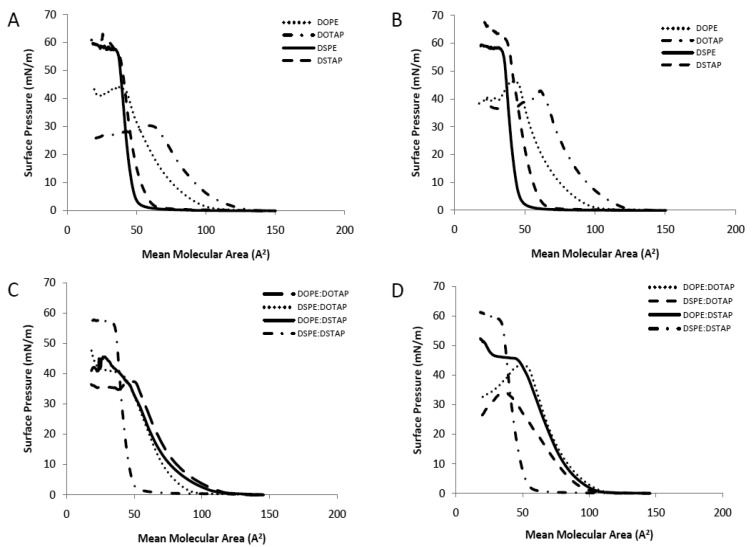
Compression isotherm studies of the pure and mixture of lipid monolayers of DOPE:DOTAP, DSPE: DOTAP,DOPE:DSTAP and DSPE:DSTAP in deionised water or PBS at 20 °C. Results are expressed as the means of three experiments. SD has not shown for clarity.

The Langmuir-Blodgett trough was used to investigate pure and mixed lipid monolayers for their interactions within the monolayer in the aqueous sub-phase of either dH_2_O or PBS to consider the translation of these studies into liposomal systems as has been previously outlined [17]. Pressure-area (π-A) isotherms are shown in Figure 2. The extrapolated (to zero pressure) area per molecule and collapse pressure for the individual lipids, in either a water or PBS subphase, and also for 1:1 lipid mixtures are shown in Table 1. For the latter, the ideal extrapolated area per molecule was calculated based on taking the average area for the lipid combination, such that the calculated area could be compared to the actual area per molecule of the mixture. Deviations between the experimentally observed and the calculated ideal area may be considered as the measure of interactions between the mixed components since the experimentally observed area depends on the intermolecular forces between the lipids in the mixed monolayer. Negative deviations (where the experimental area is less than the ideal calculated area per molecule) indicate attractive interactions occurring between the lipids whilst positive deviations indicate repulsive interactions.

Considering the single component monolayers formed on dH2O, the extrapolated area per molecule for each of the 4 lipids was in the order of DOTAP > DOPE > DSTAP > DSPE (Table 1) with the cationic lipids (DOTAP and DSTAP) having a larger area per molecule than their comparable zwitterionic counterparts (DOPE and DSPE respectively; Table 1). The cationic lipids also formed liquid-expanded monolayers (Figure 2A) with lower collapse pressures than their PE counterparts (Table 1). Comparison between the saturated and unsaturated lipids, show that the saturated lipids are able to pack together closer in a solid monolayer than their unsaturated counter parts (Figure 2A) with DOTAP having approximately twice the measured molecular area compared to DSTAP (104 *vs.* 53 A^2^/molecule; Table 1). Due to their closer packaging arrangement, the saturated lipids also display a higher collapse pressure and a more rigid monolayer than their unstaturated counterparts (Table 1 and Figure 2A). Formation of these monolayers on PBS rather than dH_2_O made no notable difference in the measured area per molecule; however this leaded to an increased collapse pressure in the case of the cationic lipid monolayers, particularly in the case of DOTAP which increased from 29.5 to 42.1 mN/m (Table 1 and Figure 2B). This resulted in their being no significant difference in collapse pressures between the cationic and zwitterionic lipid monolayers when formed in PBS (Table 1).

When prepared as mixed monolayers at a 1:1 molar ratio (as is commonly adopted in lipoplexes) the extrapolated area per molecule for the combinations was in the order of DOPE:DOTAP > DOPE:DSTAP ≈ DSPE:DOTAP > DSPE:DSTAP with the combination of two unsaturated lipids gave the highest mean molecular area whilst the fully saturated mixture (DSPE:DSTAP) had a smaller mean molecular area of 46 A^2^ per molecule (Table 1), and formed a solid monolayer similar to the individual components (Figure 2). Of the 4 mixed monolayer, this monolayer also had the highest collapse pressure (52.9 ± 1.3 mN/m; Table 1). When the dH_2_O subphase was replaced with PBS, there was a notable increase in the extrapolated area per molecule for DSPE:DOTAP suggesting the presence of buffer salts was inhibiting the packaging of the monolayer yet the collapse pressures were not influenced by the change in subphase (Table 1).

Considering the deviation from ideality (Table 1), which can be used to monitor molecular interactions between the molecules in the mixed monolayers, those lipid monolayer contains either both saturated (DSPE:DSTAP) or both unsaturated (DOPE:DOTAP) lipids the deviation is minimal irrespective of the choice of subphase (Table 1) suggesting there was no condensing effect occurring in either type of monolayer. In contrast, for the monolayers combining a saturated and an unsaturated lipid (DOPE:DSTAP or DSPE:DOTAP) there is large positive deviations from the calculated mean area particularly when PBS was used as the subphase, suggesting the lipids in these mixed monolayers packed in a more expanded arrangement than was predicted, particularly when the systems were in PBS as the deviation was >30% for both DOPE:DSTAP and DSPE:DOTAP (Table 1). However these differences do not translate into changes in collapse pressures, with both mixed monolayers having the same collapse pressures in dH2O as they did in PBS (Table 1).

Saturated long chain lipids often displace strong attractive intermolecular interactions and this is supported by the small molecular area and high collapse pressure of the saturated monolayer and this might suggest that the DSPE:DSTAP combination could give a strong low permeability liposome system, as has previously been shown with water soluble drugs entrapped within vesicles [24–26]. However in the case of unsaturated lipids, these lipids are more bulky (as shown by their larger molecular area; Table 1) with a less densely packed arrangement that can cause a more permeable liposome bilayer with higher release profile of entrapped drug compared to saturated monolayers [17,27].

From these results, it would suggest that the use of a fully saturated system promotes the higher packing density of lipids with high collapse pressure and which may promote a more rigid liposome system. In contrast, liposomes formed in PBS from lipid mixtures containing unsaturated lipid(s) in the mixture (either the helper lipid or the cationic lipid) could result in liposomes with less rigid bilayers (Table 1 and Figure 2). However given lipoplexes require both stability on storage and fusogenic properties, the consideration of how such bilayer attributes translate into liposome formulation and transfection attributes was considered.

### 3.2. The Effect of Alkyl Chain and Electrolytes on the Characteristics of Lipoplexes

To investigate the lipid properties on the lipoplex attributes, we attempted to formulate SUV based on the above combinations *i.e.*, DOPE:DOTAP, DOPE:DSTAP, DSPE:DOTAP and DSPE:DSTAP SUV formulated in distilled water or PBS and mixed with plasmid DNA (gWiz™ Luciferase) at a range of concentrations (2.5, 25, 50, 100, 200 and 1600 μg). Of the four combinations, it was not possible to formulate DSPE:DSTAP liposomes, suggesting that whilst this combination can form a closely packaged solid monolayer this could not be translated into a liposomal bilayer. Similarly DSPE:DOTAP liposomes could only be formed in dH2O and not in PBS.

As mentioned, saturated lipids often display strong attractive intermolecular forces which can make hydration and dispersion in water difficult [28,29], hence the difficulty in formulating liposomes form DSPE:DSTAP. The introduction of double bonds into the lipid tail (*i.e.*, replacement of DSPE with DOPE or DSTAP with DOTAP) results in a less compact system which is easier to disperse in water [29]. The inability of DSPE:DOTAP to form vesicles in PBS might be due to the effect of salt on critical packaging parameter (CPP) of the lipid which is depended on length of hydrocarbon chain, volume of hydrophobic part and the surface area per molecule [28,29]. Dispersion of lipid molecules in the water may lead to different structures such as micelles, inverted micelles, hexagonal, lamellar or cubic phase as well as liquid crystalline with CPP being a useful predictor of the structures formed. The desired CPP shape for lipids to form liposomes is a truncated cone shape (a CPP of between ½ and 1) however studies show that liposomes can form from lipids which individually do not have the truncated cone shape, but when combined are able to appropriately package [28–30]. In the case of DSPE:DOTAP, in water the molecular shape of DOTAP (with its large tail area and cationic head-group) may be able to compensate for the smaller DSPE molecular volume, however in the presence of buffer this can reduce the electrostatic nature of the DOTAP headgroup [29], therefore changing the ‘shape’ of the molecule which means it is not able to compensate for DSPE thus prohibiting the formation of liposomes.

#### 3.2.1. Vesicle Size and Zeta Potential of Liposomes and Lipoplexes

**Figure 3.**
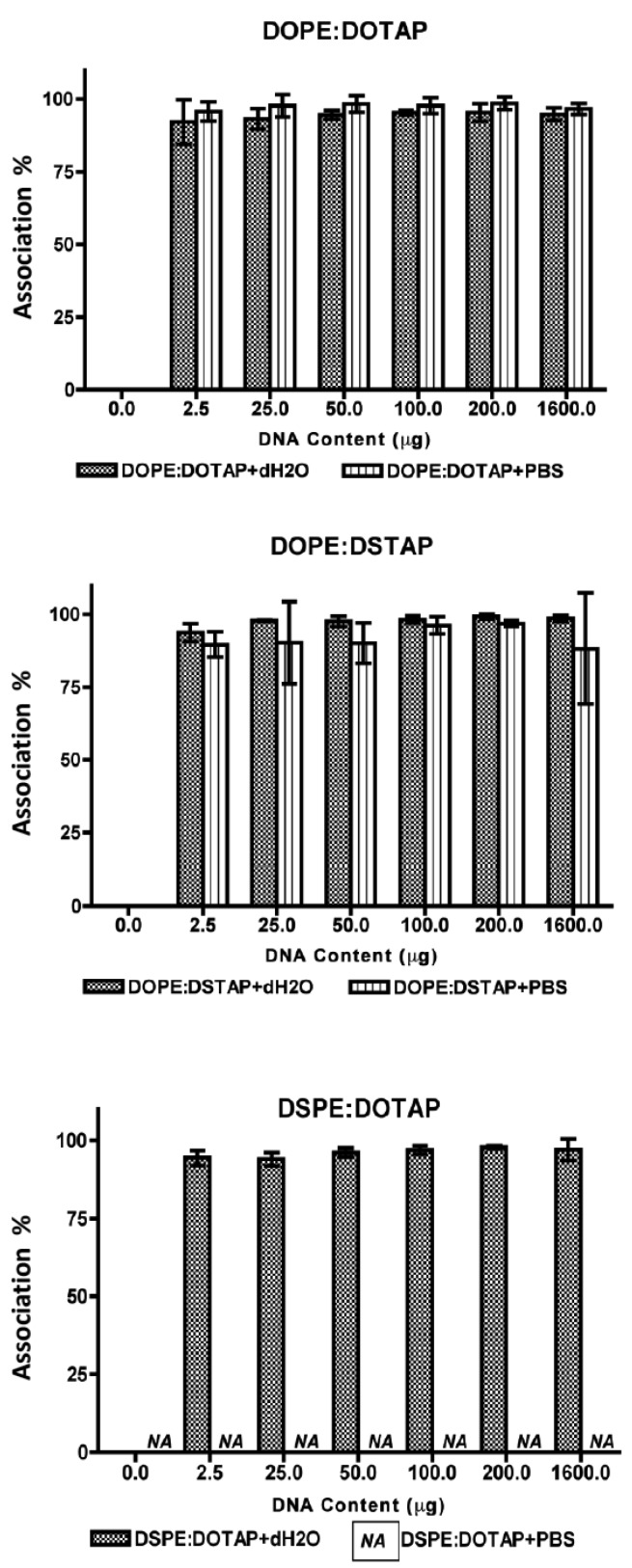
DNA association within lipoplexes of DOPE:DOTAP, DOPE:DSTAP, DSPE:DOTAP in distilled water and PBS and in various concentrations of DNA. Results denote mean ± SD, n = 3.

**Figure 4.**
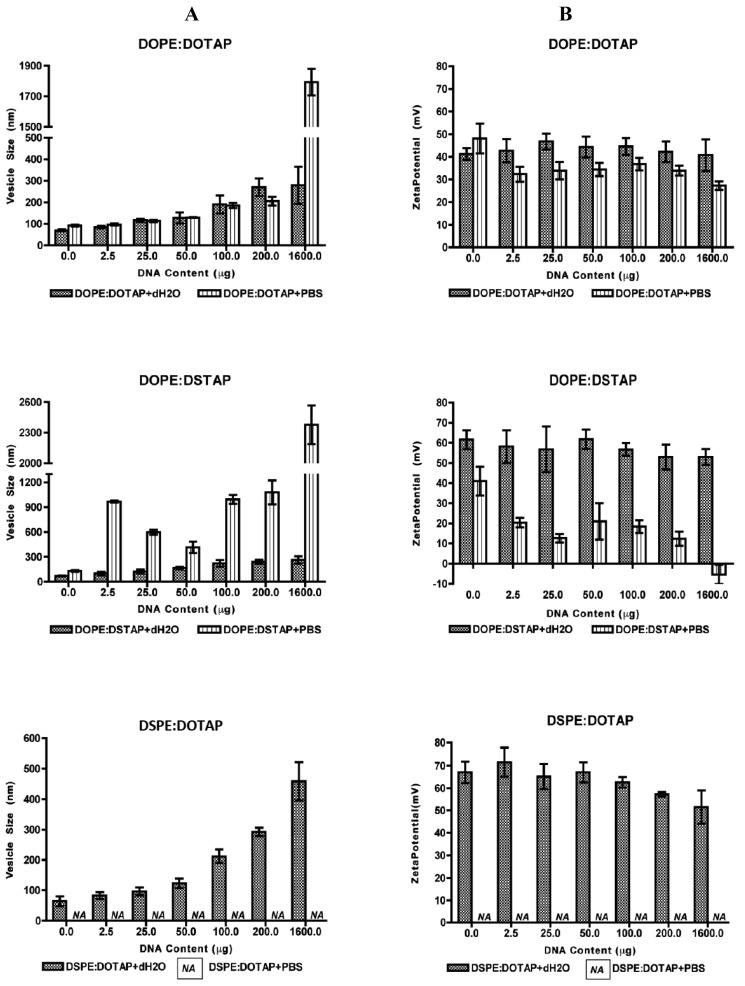
Vesicle size (**A**) and Zeta potential (**B**) of DNA lipoplexes of DOPE:DOTAP, DOPE:DSTAP, DSPE:DOTAP in distilled water and PBS and in various concentrations of DNA. Results denote mean ± SD, n = 3.

All five of the liposome formulations gave high DNA complexation across the DNA concentration range tested (Figure 3) with % DNA association being >95% in all cases showing all systems were able to electrostatically interact with the DNA as would be expected. However the effect this complexation had on the formed lipoplexes was dependent on the lipid combination and the choice of aqueous buffer (Figure 4).

In all formulations, vesicle size increased with increasing DNA concentration suggesting aggregation of the system due to what has previously been attributed to a bridging effect [31] and/or a re-organisation of the system [32–35] which is highly dependent on the +\− charge ratio [36]. With the DOPE:DOTAP formulation, the presence of the buffer salts in PBS made no significant difference in vesicle size, except for the highest DNA concentration tested; lipoplexes were around 300 nm if formulated in water compared to ∼1800 nm when prepared in PBS (Figure 4). In the case of the DOPE:DSTAP lipoplexes, the presence of PBS was shown to significantly (p < 0.05) increase the size of the lipoplexes at all DNA concentrations and of the 4 formulations, DOPE:DSTAP in PBS gave the largest lipoplexes (Figure 4). Previous research has also shown the size of the liposomes can increase to double the size in the presence of PBS as the phosphate group of the PBS can perform as glue and increase the vesicle size by bridging the cationic polar heads of the lipid [21]. Another study reveals cationic liposomes have a higher tendency to aggregation when there is salt in the formulation due to the reduced electrostatic interactions between systems and consequently more aggregation and larger vesicles formed [37]. The difference in size between the formulations may be again due to the molecular packaging of the lipids, as more rigid assemblies, such as those formed from saturated cationic lipids, have been shown to preclude efficient re-organisation of this system, causing aggregation of large particles to form with lower transfection efficacy presumably due to reduced internalisation [29].

Zeta potential studies of the systems were used to estimate the level of interaction between negative charges of DNA and positive charges of cationic lipid [38]. As this electrostatic interaction is one of the basic components of DNA complexation, and the net charge of lipoplexes is important for overcoming cell barriers [30,39], zeta potential studies were performed to determine the surface charge of the lipoplex. Results illustrate that increasing the amount of DNA over the range used had little effect on the cationic nature of the lipoplexes when formulated in dH_2_O, presumably due to the high cationic/anionic charge ratio (Figure 4). However in PBS, the neutralising effect of the buffer electrolytes on the zeta potential can be seen, particularly for DOPE:DSTAP, where the zeta potential reduce from ≈+60 mV in distilled water formulations to ≈+30 mV and less in PBS formulations.

The electrostatic interaction between positive charges of the cationic liposome and negative charges of phosphate groups of DNA are reported as the main interaction in formation of lipoplexes [21,31]. Some other researchers add that packing properties of the lipids used in the formulation of lipoplexes may also play a role in condensation of the nucleic acid [40,41]. However, as it has been shown in Figure 4 and previously Ciani *et al.*, [31] also demonstrated that the zeta potential of the lipoplexes are close to the zeta potential value of the pure liposomes at lower DNA concentrations (P > 0.05, ANOVA). They concluded that half of cationic molecules of the liposome are involved in the electrostatic interaction. These molecules are external lipids which are located on the surface of the liposome and when the liposome has been wrapped by DNA, the internal cationic lipids remain intact [31]. The effect of the buffer electrolytes in reducing the cationic charge of the lipoplexes can be due to the additional effect of phosphate polyanion in PBS, which reduces the positive charge of cationic lipids and in some cases results in their precipitation [42] however a reduction in surface charge may be beneficial to allow appropriate DNA dissociation from the lipoplex after cellular uptake to allow the DNA to reach to the nucleus [43].

### 3.3. In Vitro Transfection Efficiency

**Figure 5.**
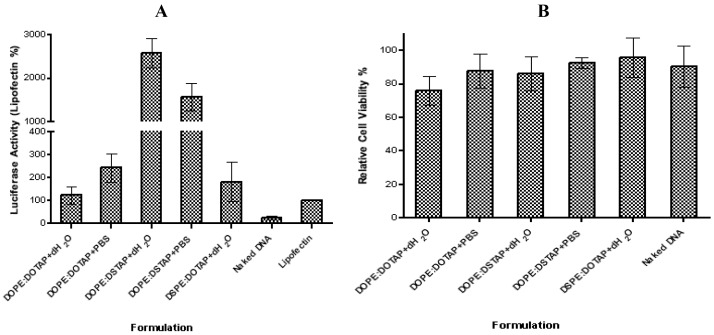
(**A**) Comparison of transfection efficiency of five cationic liposomes formulated with distilled water or PBS. Results denote mean ± SD, n = 3. (**B**) Relative cell viability of five cationic liposomes formulated with distilled water or PBS. Results denote mean ± SD, n = 3.

The five liposome formulations were tested for their ability to promote transfection, with DOPE:DSTAP in PBS showing the highest transfection levels (Figure 5). Transfection efficiency of lipoplexes formulated in water were in the order of DOPE:DSTAP > DSPE:DOTAP ≈ DOPE:DOTAP, and DOPE:DSTAP > DOPE:DOTAP when formulated in PBS (Figure 5A). This variation in transfection efficacy was not related to cytotoxicity given that at the concentrations tested there was no significant difference between the formulations (Figure 5B).

In general, decreasing the hydrocarbon chain length has been reported to increase transfection efficiency [44,45], however, given both lipids have the same carbon chain length, this case cannot be a reason for the variation in transfection efficiency shown in Figure 5. Equally the size, charge and cationic lipid/DNA ratio have all been attributed to play a role in controlling transfection [47–50] however in the case of DSTAP and DOTAP liposomes, neither the size nor the charge of the system seem to correlate to its higher transfection efficacy given it is neither obviously different in size (e.g., DOPE:DOTAP and DOPE:DSTAP in dH_2_O are similar in size but have large differences in transfection efficacy) nor significantly different in cationic nature than the other formulations (Figure 4). Similarly both systems contain the fusogenic lipid DOPE which may enhance intracellular delivery of DNA [29]. The theory of using helper lipid comes from the original research of Flenger *et al.*, in 1987 [46]. They demonstrated that the transfection activity of DOTMA when formulated with DOPE is more than when is formulated with DOPC and it has been proposed that the ability DOPE to promote the transition from lamellar phase to an inverted hexagonal phase [39] thereby promoting the conversion of the lamellar lipoplex phase into a non-lamellar structure due to the inverted cone-shaped structure of DOPE [14,44,47,48]. After endosomal uptake of the lipoplexes, the presence of DOPE in the formulation is suggested to aid distruption of endosomal membrane, allowing the release of the DNA from the endosome and the lipoplex, leaving it free it to enter the nucleus [11,31]. However in this study, lipoplexes formulated with DOPE:DOTAP and DSPE:DOTAP showed no significant difference in transfection levels suggesting that DSPE may be equally suitable as a helper lipid for unsaturated cationic lipids but not useful when combined with saturated lipids.

## 4. Conclusions

Monolayer studies on pure lipids as well as mixture of lipids showed that saturated lipids have closer packaging arrangements than their unsaturated counterparts. Whilst the use of lipids that form condensed monolayers may be beneficial in formulating low permeability bilayers, lipid combinations that form highly compact monolayers are not a suitable choice for the formulation of liposomes. In terms of transfection efficacy, neither considering the molecular packaging of the lipoplex, nor its basic physicochemical attributes, have been shown to correlate with transfection efficacy with several general assumptions being shown to be misleading in our studies, this includes the need for the fusogenic lipid DOPE to promote high transfection efficacy. Similarly, the role of electrolytes in lipoplex formulations is shown to be dependent on the formulation, with PBS diminishing the transfection of DSTAP systems yet enhancing DOTAP based lipoplexes. Therefore there remains no clear physico-chemical screening that can be adopted to predict *in vitro* efficacy. Combining this with the lack of *in vitro* and *in vivo* efficacy that plagues non-viral delivery systems suggests that for the continued development of non-viral transfection agents, new tools are needed to rationalise these differences.

## Figures and Tables

**Table 1.** The experimental extrapolated area and area compressibility of mixed and pure monolayers at the air/aq media interface at 20 °C in dH_2_O or PBS as sub-phase. Results denote mean ± SD, n = 3.

**Lipid**	**Extrapolated**		**Ideal Extrapolated**		**Deviation from**		**Collapse Pressure**	
	**Area (A^2^/Molecule)**		**Area (A^2^/Molecule)**		**Ideality (%)**		**(mN/m)**	
	**dH2O**	**PBS**	**dH2O**	**PBS**	**dH2O**	**PBS**	**dH2O**	**PBS**
DOPE	71.9 ± 6.0	70.6 ± 7.7	-	-	-	-	42.3 ± 0.4	42.2 ± 2.6
DOTAP	104.3 ± 12.9	93.4 ± 10.1	-	-	-	-	29.5 ± 1.5	42.1 ± 0.9
DSPE	47.6 ± 0.5	45.7 ± 2.3	-	-	-	-	55.7 ± 0.5	53.5 ± 1.0
DSTAP	53.2 ± 2.5	53.0 ± 2.1	-	-	-	-	50.3 ± 3.1	55.9 ± 0.8
DOPE:DOTAP	89.7 ± 6.5	81.3 ± 5.3	88.1	82.0	+1.8	-0.9	38.4 ± 1.5	42.7 ± 0.9
DOPE:DSTAP	81.6 ± 0.6	87.4 ± 1.6	62.6	61.8	+30.5	+41.4	38.8 ± 2.3	36.1 ± 2.4
DSPE:DOTAP	80.6 ± 1.5	91.7 ± 1.4	75.9	69.5	+6.1	+31.9	37.7 ± 0.3	34.5 ± 0.7
DSPE:DSTAP	46.4 ± 0.7	48.6 ± 0.2	50.4	49.3	-7.9	-1.5	52.9 ± 1.3	54.4 ± 1.2
